# Digital Diabetes Care System Observations from a Pilot Evaluation Study in Vietnam

**DOI:** 10.3390/ijerph17030937

**Published:** 2020-02-03

**Authors:** Tran Quang Khanh, Pham Nhu Hao, Eytan Roitman, Itamar Raz, Baruch Marganitt, Avivit Cahn

**Affiliations:** 1 Head of Endocrinology Department, University of Medicine and Pharmacy, Ho Chi Minh City 72000, Vietnam; khanh.tran007@yahoo.com; 2 Department of Endocrinology, University of Medicine and Pharmacy, Ho Chi Minh City 72000, Vietnam; pnhhao@yahoo.com; 3 Head Diabetes Technologies Clinic, Diabetes consultant to the Clalit Health Services, Tel Aviv 6209804, Israel; eytan@glucome.com; 4 Diabetes Unit, Department of Endocrinology and Metabolism, Hadassah Medical Center, Hebrew University of Jerusalem, The Faculty of Medicine, Jerusalem 91120, Israel; ntv502@netvision.net.il; 5 GlucoMe Ltd., Yarkona 4591500, Israel; baruch@glucome.com

**Keywords:** connected diabetes care, diabetes mellitus, glucose monitoring, digital health

## Abstract

Digital technologies are gaining an important role in the management of patients with diabetes. We assessed clinical outcomes and user satisfaction of incorporating a digital diabetes care system in diabetes clinics of a developing country. The system integrated a wireless blood glucose monitor that communicates data to any smartphone utilizing a patented acoustic data transfer method, a mobile-app, and cloud-based software that stores, analyzes, and presents data. Five hospital endocrinology clinics in Vietnam sequentially recruited all patients willing to join the study, providing they had a smartphone and access to internet connectivity. Face-to-face visits were conducted at baseline and at 12 weeks, with monthly digital visits scheduled in the interim and additional digital visits performed as needed. HbA1c levels were measured at baseline and at 12 weeks (±20 days). The study included 300 patients of whom 279 completed the evaluation. Average glucose levels declined from 170.4 ± 64.6 mg/dL in the first 2 weeks to 150.8 ± 53.2 mg/dL in the last 2 weeks (*n* = 221; *p* < 0.001). HbA1c levels at baseline and 12 weeks declined from 8.3% ± 1.9% to 7.6% ± 1.3% (*n* = 126; *p* < 0.001). The digital solution was broadly accepted by both patients and healthcare professionals and improved glycemic outcomes. The durability, scalability, and cost-effectiveness of this approach merits further study.

## 1. Introduction

Patients with diabetes generate substantial amounts of clinically significant data on a daily basis. Self-monitoring of blood glucose (SMBG) levels along with automated or patient-reported recordings of food intake, physical activity, medication, and insulin timing and doses can form an entire metabolic kaleidoscope [1,2,3]. These data often go unobserved, and the prevailing challenge is collecting and using these data to improve patient care, as well as achieving lower glucose levels.

Striving for glucose levels that are as low as can be safely attained reduces long term complications [4]. This can be achieved by monitoring glucose levels—at individually dictated intervals—and adjusting diet, medication, or insulin doses accordingly [4]. SMBG is frequently performed by patients, yet, often there is no resultant action. Patients may be unsure of if or how to modify their dietary or medical regimen in light of the glucose measurements. Moreover, physicians and other members of the healthcare team may neglect to review these data with the patients during their infrequent visits to the diabetes clinic. Additionally, several weeks following an episode of significant hyper- or hypo-glycemia, the patient may not remember what led to the abnormal glucose measurement, and cannot discuss with the healthcare team the means to prevent subsequent events from occurring [5].

Aiming to utilize patients’ self-monitored blood glucose data, a point of care glucose monitoring device combined with mobile technology can enable real-time transmission of glucose values to a centralized system [6,7,8]. Supplementing this system with automatic alerts that can be transmitted to the healthcare providers, patients, or significant others, can enable mindful and timely use of these data. Furthermore, centrally available data may be used to monitor a single clinic or a large population, follow patient trends, and recognize regional patterns. This may enable reaching out in time to patients who are in need, while reducing the frequency of visits of patients who are well-controlled, thus enabling judicious allocation of limited time resources.

The GlucoMe digital diabetes care platform is designed to address these multiple aspects of diabetes care for patients, caregivers, healthcare professionals, and providers/payers. The platform integrates a novel glucose monitoring device, mobile software, and a cloud-based big data computing infrastructure in order to close the loop of diabetes care, improve outcomes, and streamline costly diabetes management [9]. The simplicity of the device, mandating only that the user has a smartphone and internet connectivity, while precluding the need for manual download of the data, enables its incorporation in diabetes clinics of a developing country.

Diabetes care is ever more challenging in developing countries, with diabetes prevalence soaring in the face of limited healthcare personnel and resources. Nevertheless, smartphone connectivity is high, with 84% of the people in the major cities, and 68% in rural areas overall owning a smartphone in Vietnam [10]. This may present an opportunity for increasing access to diabetes care.

This pilot study was designed to evaluate the market acceptance of GlucoMe’s digital diabetes system in Vietnam and to assess the feasibility of incorporating it in the clinical care of patients with diabetes in a developing market.

## 2. Materials and Methods

### 2.1. The Digital Diabetes Care System

**Figure 1:**
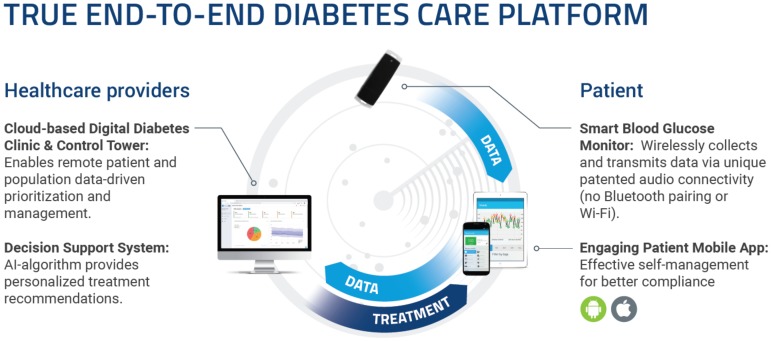
System components.

The digital diabetes care system was developed by GlucoMe to provide a holistic digital solution for patients with diabetes and their healthcare providers. It is composed of several components (Figure 1):

(1) *Wireless point of care blood glucose monitor*. This CE (Conformité Européenne )-certified blood glucose monitor (BGM) is not dependent on traditional wireless communication protocols but connects to any iOS or Android smartphone using a simple, unique, and patented acoustic data transfer method. Data in the BGM are converted to binary format and encoded to audio frequencies by a proprietary audio protocol along with an error correction mechanism. These are transmitted through the BGM’s speaker and received by a smartphone’s microphone. The smartphone parses the audio waves and converts them to binary and then to actual values. The glucose monitor’s data transmission mechanism is not influenced by environmental noise. Tested in over 1200 scenarios at various noise levels and diverse environments (office, concerts, nature, restaurants, etc.), it performed flawlessly, with 100% measurement accuracy and integrity [9].
(2) *Mobile app.* The app is compatible with any iOS or Android smartphone, and securely transmits real-time blood glucose data for cloud-based analyses, enabling two-way communication between patients and healthcare professionals. The app provides a set of user-friendly monitoring and management tools for the patient, including a logbook of measurements, estimated HbA1c, glucose distribution, and trends. It additionally enables data sharing with caregivers and medical professionals, formulation of diabetes treatment plans, as well as carbohydrate intake and exercise tracking.
(3) *Cloud-based digital diabetes clinic and control tower software.* This software can utilize data from the blood glucose monitor, or connect to external sources for data. It analyzes and presents the patients’ data in a user-friendly interface. Data can also be collated from multiple patients and clinics to provide population management reports. Individualized alerts can be defined according to pre-specified thresholds and sent to patients or healthcare professionals.

The system received regulatory approval in Vietnam and, prior to the study, the entire system was adjusted to the Vietnamese market, including translations of the smartphone app, digital diabetes clinic, and control tower software, as well as blood glucose monitoring instruction for use, along with video and commercial materials.

### 2.2. Study Design

This pilot study was a single arm market acceptance evaluation study aiming to assess the feasibility of incorporating a digital diabetes care system into routine patient care in a developing country. The study assessed changes in glycemic parameters, as well as patient and physician satisfaction with the system.

The study enrolled patients in five endocrinology clinics in Ho Chi Minh City (HCMC), Vietnam, between August 2018 and January 2019. The study was approved by the hospitals’ ethics committee, and all patients signed informed consent prior to participation.

### 2.3. Study Conduct

#### 2.3.1. System Setup

Prior to study initiation, the GlucoMe software system (GlucoMe Ltd. Yarkona, Israel) was installed in the hospitals’ endocrinology clinics. Two group orientation sessions for the healthcare professionals were held, followed by individual face-to-face meetings and remote support sessions, on a per-needed basis. Ongoing technical support to the healthcare professionals as needed was provided by dedicated local support team members.

#### 2.3.2. Inclusion Criteria

Patients were recruited in the endocrinology clinic of each participating hospital, on the basis of the natural flow of patients in the clinic. All patients attending the clinic during the study period who could benefit from regular glucose monitoring as judged by their treating physicians were offered participation. Patients were included, providing they (1) had a smartphone and were able to operate the device, and (2) had ongoing access to internet connectivity.

#### 2.3.3. Enrollment Visit

Patients suitable for the pilot signed informed consent forms prior to participation. Each patient then received a blood glucose monitoring kit and the GlucoMe App was downloaded and installed on the patient’s smartphone. Training on the operation of the blood glucose monitor and smartphone application was provided, and it was based on a detailed patient onboarding protocol. A demo test was conducted on-site by the patient to ensure the patient’s understanding on the mode of operation. The preferred interaction mode with the patient was discussed and agreed upon (Short Message Service (SMS) texting, telephone, etc.), to enable smooth communication in the ongoing digital visits. The healthcare professional defined the individualized thresholds for automatic messages to be sent to the patients, offering positive feedback or prompting action. Blood was drawn for HbA1c, unless a recent HbA1c result (within 20 days) was available.

On average, the patients’ onboarding process took about 30 min. Group orientation sessions were performed for most of the patients in groups of three to four patients.

#### 2.3.4. Digital Visits

**Figure 2:**
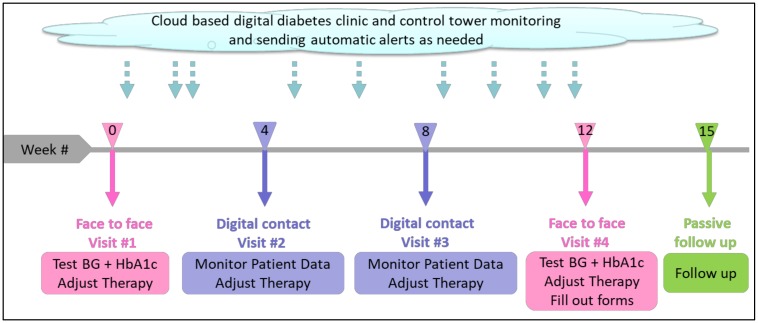
Study design.

Digital visits were conducted monthly, following the initial visit, and additional digital visits were performed as needed (Figure 2). These visits constituted a review of the patient’s glucose log and analytics using the digital diabetes clinic desktop application. On the basis of the data, the physician determined whether the existing treatment was appropriate or if a treatment change was mandated. Upon completion of a digital visit, a summary message was sent to the patient via the digital diabetes clinic system. Unscheduled digital visits were added as needed to address patient queries or control-tower alerts that prompted for action.

#### 2.3.5. Patient Data Monitoring and Automated Alerts

The digital diabetes clinic control tower system software was operated mainly by a healthcare professional (a doctor or a nurse) who monitored all patients connected to the system in a routine ongoing fashion. Alerts were generated automatically on the basis of each patient’s glycemic control targets and SMBG adherence alert definitions. For example, an alert could be generated in response to a single or several glucose measurement crossing a pre-specified threshold, or in response to lack of adherence to the predefined SMBG protocol in the recent week. Each alert was addressed through a resolution protocol, which was based on clinical instructions of the medical staff communicating with the patient using pre-defined messages of the “Hospital customized messaging library”. Some alerts mandated getting in touch with the patient in the mode that was agreed upon at enrollment, whereas others consisted of sending the patient a reminder for glucose monitoring or offering positive feedback.

#### 2.3.6. Final Visit

The patient returned to the clinic for a final visit 12 weeks after enrollment. The patient’s treatment regimen was reviewed and modified. Blood was drawn for HbA1c unless a recent (within 20 days) HbA1c test result was available and was used as an end of pilot result. The patient completed a satisfaction questionnaire regarding ease of use of the glucometer (5 questions), mobile app (10 questions), and overall system (4 questions). The treating physician completed a satisfaction questionnaire regarding personal experience with the GlucoMe system in the management of the specific patient. Physicians completed satisfaction questionnaires following the entire trial period as well. Full questionnaires are available in the online Supplementary Materials. Patients were followed for an additional 20 days for adverse events and ad-lib glucose monitoring.

### 2.4. Outcome Measures

Outcome measures included:

(1) Change in glycemic parameters—overall glucose levels, fasting glucose levels, and HbA1c.
(2) Frequency of glucose monitoring.
(3) Patient and physician satisfaction as assessed by questionnaires.

We also assessed the frequency of contact between patients and healthcare professionals, defined as digital touchpoints. A day with a “digital touchpoint” is a day in which at least one of the following happened: a control tower event was resolved by the medical team, a digital visit took place, or a message was sent from/to the patient to/from the medical team.

### 2.5. Statistical Analyses

Only patients completing the pilot were included in the data analyses. Data presented are mean ± SD. Difference in overall glucose levels between initial 2 weeks and final 2 weeks was calculated only for those patients with glucose measurements at both time points. Glucose levels that were tagged by the patient as fasting were included in analysis of fasting glucose levels.

The average responses to each aspect of the system were calculated for all patients and are presented as averages of the categories. The responses of the physicians to the individual questions were calculated as well. Obstacles to care were listed in the satisfaction questionnaires as free text, and these were grouped by two members of the research team, each individually, with disagreements resolved by discussion. SPSS Statistics version 25.0 (IBM, NY, USA) was used for all analyses.

## 3. Results

**Figure 3:**
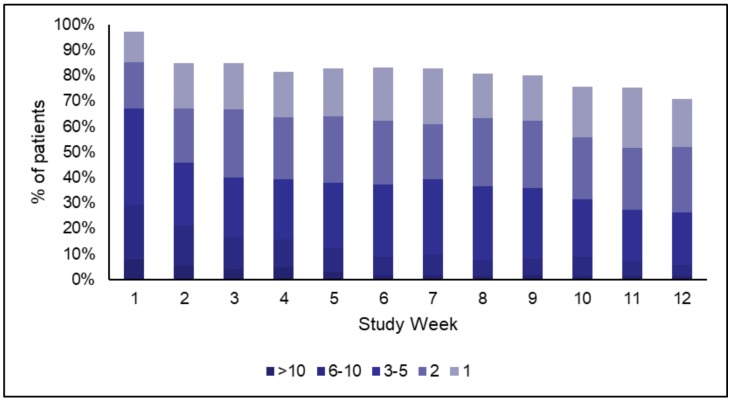
Weekly glucose measurements.

Overall, 300 patients were enrolled in the pilot in 5 participating hospitals, of whom 279 completed the evaluation. Dropout was due to change in internet access availability (18) or death (3). The average number of weekly glucose measurements is shown in Figure 3. At week 12 and during the 20-day follow-up period afterwards, 81% of the patients were still engaging with the system and had still been measuring glucose.

**Figure 4:**
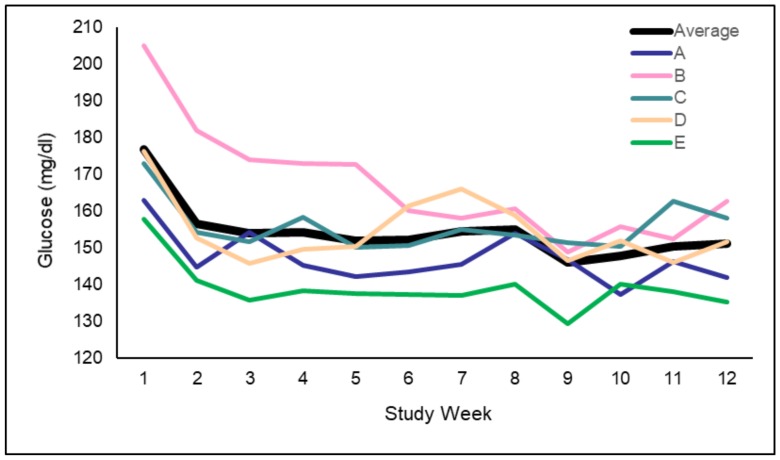
Average glucose levels in study weeks by participating centers.

Average glucose levels, as seen in Figure 4, declined from 170.4 ± 64.6 mg/dL in the first 2 weeks to 150.8 ± 53.2 mg/dL in the last 2 weeks (*n* = 221, *p* < 0.001). Fasting glucose levels declined from 141.9 ± 39.3 to 135.1 ± 32.5 (*n* = 103, *p* < 0.001). HbA1c levels at baseline and 12 weeks were available for only 126 of the patients, and declined from 8.3% ± 1.9% to 7.6% ± 1.3% (*p* < 0.001).

Over the study period, an average of 14 digital touch point days occurred per patient, equivalent to an average of 1 digital touchpoint every 6.2 days.

There was significant variation in the average number of digital touch point days between hospitals, spanning from 9–18 days in the different centers.

Overall, 262 patients completed the questionnaires at the final visit (Table 1). Patients were satisfied with the technical aspects of the glucometer, the mobile app, and the overall system. There were 23 physicians who completed questionnaires regarding 260 individual patients and regarding their overall satisfaction with the system. Physicians felt the system led to better care, efficiency, and organization of the data, and would recommend using it (Table 1).

**Figure 5:**
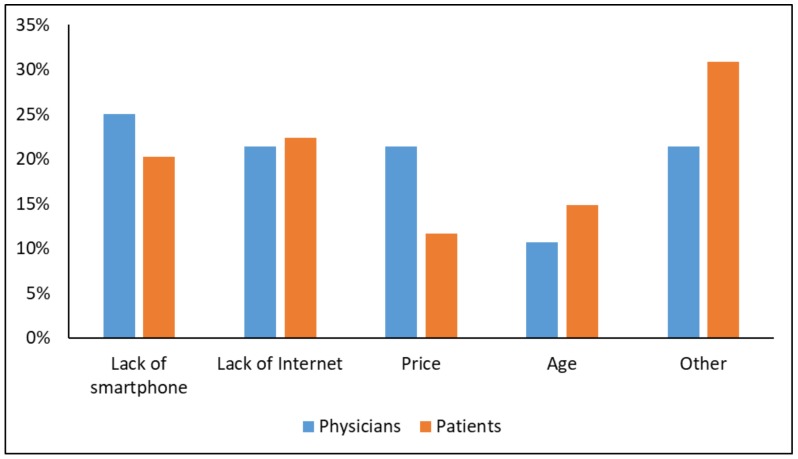
Obstacles to global implementation of the system.

The physicians and patients listed possible obstacles for expanding the GlucoMe service in Vietnam as free text, and answers were grouped by the pilot team. Leading obstacles noted included lack of smartphone, lack of internet connectivity, and cost. Other obstacles mentioned included limited availability, older age, requirement for training, and fear of acceptance of a new technology (Figure 5).

## 4. Discussion

In this pilot study, we demonstrated the feasibility of incorporating digital technology in the care of patients with diabetes in a developing country. Glucose levels declined within the first 2 weeks, with a durable effect throughout the study. Additionally, the vast majority of patients remained engaged with the system over the study time, and a clinically significant HbA1c reduction was obtained. Moreover, patient and physician satisfaction with the system overall and its components was high.

SMBG has become an important aspect of patient care because of its widespread availability. This is most evident for patients who are taking insulin, where SMBG is the basis for dose titration; however, even in non-insulin-treated patients, SMBG may facilitate better adherence to dietary and medication regimen. In standard practice, SMBG values are often ignored in the routine clinical visit, overlooking clinically significant data, which may assist in decision making [5]. Studies examining the glycemic benefits of SMBG have shown mixed results, whereby a major determinant of benefit was the extent to which patient education, as well as response to SMBG levels, was implemented [11,12,13]. A meta-analysis of studies that utilized automatic mobile transmission of SMBG levels showed a HbA1c benefit of −0.27 (95% CI: −0.03, −0.51) [14]. However, when analyzing studies by mode of delivery of telemedicine, that is, by human calls or human messages (SMS or online), these led to HbA1c reductions of −0.98 (−0.42, −1.54) and −0.69 (−0.26, −1.13), respectively [14]. A meta-analysis of interventions incorporating individually tailored text messaging, with many messages addressing uploaded glucose values, demonstrated a meaningful 0.54% (95% CI: −0.08, −0.99) HbA1c reduction [15]. Thus, SMBG prescription to patients should be a part of a broad educational program, with ongoing instruction and regular evaluation in order to benefit from its use and justify it economically [7,11]. In our study, GlucoMe’s digital diabetes system streamlined SMBG readings and utilized both automated messaging as well as human calls or messages, which were scheduled monthly with additional contact made as needed. This judicious use of the frequently overlooked SMBG levels may have contributed to the reduced glycemic levels observed in our study, although improved adherence and medical follow-up were probably important contributors as well.

Digital technologies are gaining an important role in the management of patients with diabetes, enabling close monitoring and advanced follow-up of patients’ glycemic control and adherence to treatment. Nevertheless, over the decade of evolvement of these novel technologies and approaches, it is becoming clear that although digital health may facilitate better care, it will not replace the human touch. Human factors such as empathy, compassion, and experience cannot be fully captured and taken into consideration in the digital interactions, and inter-personal contact will remain an essential part of the patient visit [16].

In this sense, the GlucoMe digital diabetes system creates a balance that preserves yet supplements the human factor. The limited time window of the encounter of the patient with the healthcare professional is not wasted on analyzing or recording data. These are presented conveniently to the caregiver by utilizing the digital diabetes system software, thereby enabling a discussion that is focused on that which is beyond the data. The initial and final study visits in our study were a face-to-face interaction, yet, the interim digital touch-points presented an opportunity to fine-tune care and retain some therapeutic tension during the study. This combined approach may have contributed to the good durability observed in our study.

Interestingly, although we observed a gradual decline in patients’ engagement with the system, glucose levels declined early and remained low, with a resultant improvement in HbA1c levels. This may indicate a possible “learning curve” of the patients, who adjusted their lifestyle and medication regimen according to the more frequent initial readings, and successfully adhered to the changes these mandated over time. Moreover, interaction with the system occurred in average on a weekly basis, with a digital touchpoint every 6.2 days; thus, in spite of reduction in measurements, patients still remained engaged with the system to some extent.

Overall satisfaction with the system was high, as reflected both in the patient and physician questionnaires. The patients found the system easy to use, with high satisfaction rates found especially for the glucose monitor, whereby its connectability features did not hamper ease and simplicity of use. Physicians felt the use of the system simplified data intake and led to greater efficiency and better care. With the use of the system, the physicians were able to proficiently review the patients’ data, which was displayed in a graphical and self-explanatory manner, leading to more efficient use of time during the clinical encounter. Moreover, the system enabled selection of patients in poorer control—mandating unscheduled digital visits to improve glycemia, and yielding better use of limited time. Although uploading and installing the system was time consuming, it did pay off in better use of clinical time thereafter. Although cost was listed by some as a barrier to further expansion of this technology, time saving obtained by precluding the need to download data and by faster interpretation of data may lead to cost saving. Still, the cost of SMBG—that is, the actual cost of the BGM, and particularly of the testing strips—comprise the majority of the cost burden, and this remains an economic challenge in developing countries.

Connected glucose monitors have become increasingly popular in recent years [7,17,18,19]. These aim to provide continuous care, increase patient satisfaction, and improve glycemic control while leading to cost-saving. The majority of these applications demonstrated clinical efficacy, mostly on the basis of observational data, and some on randomized controlled studies. Reported improvements in HbA1c ranged from 0.3–2.4%, with most reductions at <1.0%, with baseline HbA1c being an important determinant of change [7]. In this respect, the preliminary results of our study showing an HbA1c reduction of 0.7% is in line with published data. Additionally, similar to what has been observed in our study, connected glucose meters led to improved patient satisfaction [7,18]. Reduced medical spending has been demonstrated in a large study of connected glucose monitoring [20], and additional cost-effectiveness data is being collected for many other applications as well [7]. Nonetheless, in spite of the wealth of data available on connected care in developed countries, the feasibility of its implementation in developing countries is less well established. Telemedicine has been employed in developing countries for multiple aspects of diabetes care. It has been utilized to increase awareness to the disease and its complications and to monitor glucose levels. In addition, some studies have been conducted on remote screening for retinopathy, although further study of the feasibility and cost-effectiveness of these different approaches mandate further study [21]. In our study, approximately a quarter of the patients and physicians felt lack of smartphone or internet connectivity would be significant obstacles in further promotion of the technology in Vietnam. The patients joining our study did not have these technological limitations (excluding 6%), and thus may not be representative of the overall diabetic population in Vietnam. However, with the expanding use of internet and smartphones in Vietnam and other developing nations, this issue will probably be further mitigated in the upcoming years.

Several limitations of our study should be noted. First, this was a single-arm market evaluation pilot study, and thus the participating population may have high motivation and competence, not reflective of the overall diabetic population. Second, the study duration was short, and although the preliminary data are promising, long term follow-up is mandated. Finally, due to local regulations, patient identified data were collected and retained locally, and only glucose levels and data gathered digitally during the study were centrally analyzed.

## 5. Conclusions

In conclusion, this market evaluation study established the feasibility of incorporating digital care in the routine care of patients with diabetes in a developing market. Glycemic indices improved and patient and physician satisfaction with the system were extremely high.

This study indicates the existing potential in implementation of digital technologies in emerging markets, which despite limited resources are capable of harnessing mobile technology to diabetes care. Nevertheless, putting this program into practice at a large scale requires long-term follow up and cost-effectiveness analyses. A future longer and controlled study will assess the effect of the intervention compared to standard-of-care on the glycemic effects observed. Moreover, a sub-study will ascertain the resources required for program set-up vs. long-term cost-saving derived from improved and targeted care.

## Figures and Tables

**Table 1:** Patient and physician satisfaction.

Questionnaire Item	Extremely Satisfied	Very Satisfied	Satisfied	Very Dissatisfied	Extremely Dissatisfied
	5	4	3	2	1
**Patient satisfaction (*n* = 262)**					
**Overall system** (5 questions)	54%	42%	4%	0%	0%
**Mobile app** (10 questions)	37%	28%	34%	1%	0%
**Blood glucose monitor** (4 questions)	40%	33%	26%	1%	0%
**Overall physician satisfaction (*n* = 23)**					
**Better organization of data**	52%	48%	0%	0%	0%
**Better efficiency**	65%	35%	0%	0%	0%
**Better care**	52%	43%	4%	0%	0%
**I would recommend using it**	48%	48%	4%	0%	0%
**Physician satisfaction per patient (*n* = 260)**					
**Ease of using app**	44%	40%	16%	0%	0%
**Ease of reading glucose**	42%	43%	14%	0%	0%
**Ease of reading reports**	43%	42%	15%	0%	0%
**Usefulness of data**	39%	39%	21%	1%	0%
**General impression**	44%	38%	18%	0%	0%

## Supplementary Materials

The following are available online at https://www.mdpi.com/1660-4601/17/3/937/s1, File 1: Patient and Physicians Satisfaction Questionnaires.
