# Supplementary material for: Digital Diabetes Care System Observations from a Pilot Evaluation Study in Vietnam
Source: Int J Environ Res Public Health. 2020 Feb 3;17(3):937. doi: 10.3390/ijerph17030937 (PMC7037177; doi:10.3390/ijerph17030937)
Supplement: Supplementary file 1 [file ijerph-17-00937-s001.pdf]

Supplementary material

1. Patient Satisfaction Questionnaire – patients answered all questions on a scale of 5-1 (extremely satisfied – extremely dissatisfied). Averages of results for all questions regarding each aspect of the system were calculated.
   Obstacles were listed as free text.
2. Physician Satisfaction Questionnaire – Per Patient - physicians answered all questions on a scale of 5-1 (extremely satisfied – extremely dissatisfied). Average of results for each question was calculated.
3. Physician Satisfaction Questionnaire – Group Assessment - physicians answered all questions on a scale of 5-1 (extremely satisfied – extremely dissatisfied). Average of results for each question was calculated.
   Obstacles were listed as free text.

Patient Satisfaction Questionnaire

1. Please rate your satisfaction of using the GlucoMe glucometer for glucose test with regards to the following aspects:
    1) Ease of positioning the test strip in the GlucoMe glucometer
    2) Ease of placing the blood glucose sample on the test strip
    3) Duration of time to insert the strip into the glucometer and get the results on the application
    4) Ease of using the GlucoMe glucometer as compared to your previous device
    5) Duration of the entire process of sugar blood test

2. Please rate your impression of using the GlucoMe application on your mobile with regards to the following aspects:

    1) Ease of accessing the application on the mobile
    2) Ease of reading the information in the application
    3) Ease of reading your glucose results in the application
    4) The order that the information appears in the application
    5) Ease of reading your blood glucose reports in the application
    6) Ease of understanding the error or any other messages in the application
    7) Ease of understanding the reports on your glucose levels in the application
    8) Usefulness of the information other than the glucose values
    9) Ease of entering information (such as injection, carbohydrate consumption) to the application
    10) General impression of the application

3. Based on your experience using the system, please rate your impression on the GlucoMe system with regards to the following statement:
    1) Through the GlucoMe app, I feel better connected to my physician
    2) Through the GlucoMe app, I feel better monitored and taken care of
    3) Through the GlucoMe app, I have a better trust in the treatment decisions of my physician because it is data-driven
    4) I would strongly support the broad usage of the GlucoMe platform across hospitals

4. What is, in your opinion, the biggest obstacle to more people using GlucoMe in your country?

Physician Satisfaction Questionnaire – Per Patient

Please rate your impression of using the GlucoMe application with regards to the following variables and with the impression based on the treatment with this specific patient:

1) Ease of accessing the application
2) Ease of reading the glucose levels data
3) Ease of reading the reports
4) Usefulness of the data and the reports other than the glucose values
5) General impression from the application

Physician Satisfaction Questionnaire – Group Assessment

1. Please rate your impression on the utility / value of GlucoMe system with regards to the following statements and with the impression based on the treatment of the group of patients you have followed with the system

    1) GlucoMe platform helped me better organize my patients
    2) GlucoMe platform helped me to be more efficient and allocate more time to those patients who need a Face-to-Face consultation vs. patients who I can reach out digitally
    3) By using the GlucoMe platform, patients are receiving a better care because I can better organize them based on their needs, make data-driven treatment decision and intervene in real-time
    4) I would strongly support the broad usage of the GlucoMe platform across hospitals.

2. What is, in your opinion, the biggest obstacle to more people using GlucoMe in your country?
